# *Gardnerella* species exhibit synergy in their ability to displace *Lactobacillus crispatus* adhered to HeLa cells

**DOI:** 10.7717/peerj.20076

**Published:** 2025-11-05

**Authors:** Ângela Lima, Joana Castro, Christina A. Muzny, Nuno Cerca

**Affiliations:** 1 Laboratory of Research in Biofilms Rosário Oliveira (LIBRO), Centre of Biological Engineering (CEB), University of Minho, Braga, Portugal; 2 LABBELS - Associate Laboratory, Braga, Portugal; 3 Instituto Nacional de Investigação Agrária e Veterinária (INIAV), Vairão, Portugal; 4 Division of Infectious Diseases, University of Alabama at Birmingham, Birmingham, AL, United States of America

**Keywords:** Bacterial vaginosis, *Gardnerella spp.*, Lactobacillus crispatus, Microbial synergisms, Polymicrobial biofilm, Pathogen competition, Quantitative PCR (qPCR), HeLa cell adhesion assay, Hypothesis testing in microbiota research, Lactobacillus displacement

## Abstract

**Background:**

Bacterial vaginosis (BV) is the most common vaginal infection in reproductive-age women. It is associated with adverse pregnancy complications, such as preterm birth and low birth weight, in addition to an increased risk of acquisition of HIV and sexually transmitted infections. BV is characterized by a vaginal dysbiosis, involving loss of protective *Lactobacillus* species (including *L. crispatus*) and overgrowth of facultative and strict anaerobic bacteria, with *Gardnerella* species playing a predominant role. However, despite extensive research on BV pathogenesis, its etiology remains unclear, and the sequence of events leading to the displacement of lactobacilli by anaerobic bacteria in women has not yet been fully elucidated. Until 2019, all bacteria belonging to the *Gardnerella* genus were considered part of the species *G. vaginalis*. However, it is now recognized that different *Gardnerella* species exist, each with varying virulence potentials. Recent data have shown that multiple subgroups of* Gardnerella* spp. are frequently detected simultaneously in the vaginal microbiota of women with BV. With this in mind, we aimed to test the hypothesis that different combinations of known *Gardnerella* species, isolated from the vaginal microbiota of women with BV, have an enhanced ability to compete against *Lactobacillus crispatus*, pre-adhered to HeLa cells, thereby facilitating the early stages of BV development.

**Methods:**

Adhesion assays of dual combinations of *Gardnerella* spp. (*G. vaginalis*, *G. leopoldii*, *G. swidsinskii*, *G. piotii*) were performed on an *in vitro* model of HeLa cells, covered with and without *L. crispatus*. Quantification of the species used in our assays was subsequently performed using quantitative polymerase chain reaction (qPCR).

**Results:**

Our results revealed synergy between different *Gardnerella* spp., demonstrating their ability to overcome the presumed protective effect of *L. crispatus*, thereby creating favorable conditions for the development of a polymicrobial biofilm characteristic of BV. The combination of *G. vaginalis* and *G. leopoldii* showed the greatest synergistic effect on initial adhesion to HeLa cells while the combination of *G. leopoldii* and *G. swidsinskii* had the greatest ability to reduce *L. crispatus* colonization.

**Conclusions:**

Although this *in vitro* study does not unequivocally prove that BV is initiated by the disruption of normal vaginal microbiota by *Gardnerella* spp., it strongly supports this possibility, contributing to a better understanding of BV etiology.

## Introduction

Bacterial vaginosis (BV) is the most common vaginal infection in reproductive-age women. It is associated with multiple adverse health outcomes, including infertility, pregnancy complications such as preterm birth and low birth weight (Sethi et al., 2025), in addition to increased risks of HIV and sexually transmitted infection (STI) acquisition, pelvic inflammatory disease, and cervical cancer in the setting of HPV-coinfection (Muzny et al., 2022). Microbiologically, BV is a dysbiosis of the vaginal microbiota, characterized by loss of protective *Lactobacillus* spp. and an increase in facultative and strict anaerobic bacteria, such as *Gardnerella* spp., *Fannyhessae vaginae*, *Prevotella* spp., and *Mycoplasma hominis,* among others (Muzny & Sobel, 2023). During BV, a multi-species biofilm is formed on the vaginal epithelium, with *Gardnerella* spp. thought to be pivotal in the initial stages of biofilm formation (Abou Chacra, Fenollar & Diop, 2022). However, the mechanisms leading to the development of this infection are not fully understood (Redelinghuys et al., 2020). Because BV has a high prevalence and is linked to a high rate of recurrence following treatment, further research regarding its etiology is of utmost importance (Muzny et al., 2022).

We previously hypothesized that BV development is initiated by virulent *Gardnerella* spp. that displace protective *Lactobacillus* spp. from the vaginal epithelium. Then, those virulent *Gardnerella* spp. may initiate the formation of the polymicrobial biofilm (Muzny et al., 2019). Data to support this hypothesis are derived from the observations that *Gardnerella* spp. have a significantly higher ability to adhere to HeLa cells compared to 29 other BV-associated bacteria (BVAB) (Alves et al., 2014), and that *Gardnerella* spp. isolated from BV cases have a significantly higher ability to displace lactobacilli pre-adhered to HeLa cells compared to *Gardnerella* spp. isolated from healthy women (Castro et al., 2013; Castro et al., 2015). In 2019, the description of the *Gardnerella* genus was emended and three new species, isolated from the vaginal microbiota, were proposed in addition to the previously identified *Gardnerella vaginalis*: *Gardnerella leopoldii, Gardnerella piotii*, and *Gardnerella swidsinskii* (Vaneechoutte et al., 2019). Although multiple subgroups of *Gardnerella* spp. are frequently detected simultaneously in the vaginal microbiota (Hilbert et al., 2017), there is no research on their interactions when adhering to vaginal epithelial cells. With this in mind, we hypothesized that frequent vaginal co-colonization by different *Gardnerella* spp. is the result of synergistic interactions that occur in the early stages of BV development, namely, in the initial adhesion to the vaginal epithelium. To test our hypothesis, we devised an experimental model, where pair-wise combinations of *G. vaginalis*, *G. piotii*, *G. leopoldii* and *G. swidsinskii* challenged pre-condition HeLa cells colonized by *Lactobacillus crispatus*, a key indicator of vaginal health (Huang et al., 2014).

## Materials & Methods

### Growth conditions and strains

*G. vaginalis* UM 121, *G. leopoldii* UGent 09.48, and *G. swidsinskii* CCUG 44005 were isolated from women with BV, *G. piotii* UGent 18.01 was isolated from a woman with intermediate microbiota (Nugent score 4–6), and *L. crispatus* CCUG 44128 was isolated from a healthy woman. *L. crispatus*, *G. vaginalis*, *G. piotii*, *G. leopoldii,* and *G. swidsinskii* were grown on Columbia blood agar base (CBA) (Oxoid, Basingstoke, UK) supplemented with 5% (v/v) defibrinated horse blood (Oxoid), at 37 °C under 10% CO_2_. *Escherichia coli* ATCC 25922 was also grown on CBA, but in an aerobic atmosphere at 37 °C. The inocula were cultured in New York City III broth supplemented with 10% (v/v) inactivated horse serum (Biowest, Nuaill’e, France) for 48 h at 37 °C, under 10% CO_2_ in static conditions (Castro et al., 2015). The optical densities used for all *Gardnerella spp.* was 0.18 which corresponds to a bacterial concentration of 1  × 10^8^ CFU/mL, and for *L. crispatus* was 0.13 that corresponds to 5 × 10^7^ CFU/mL, as previously demonstrated (Rosca et al., 2022; Lima et al., 2024).

### Adhesion assays

Human cervical HeLa cells (from the American Tissue Culture Collection, ATCC CCL-2) were cultured in Dulbecco’s Medium (Pan Biotech, Aidenbach, Germany) supplemented with 15% (v/v) Fetal Bovine Serum (FBS; Pan Biotech) and 1% (v/v) antibiotic (ZellShield^®^, Berlin, Germany) at 37 °C and in 5% CO_2_. Cells were cultured in 24-well tissue culture plates (Orange Scientific, Braine L’Alleud, Belgium) for 48 h. Before the displacement and adhesion assays, the confluence of the cells was verified with an inverted microscope (Leica DM IL). Next, the HeLa cells were washed with one mL of 1 × PBS to remove the non-adherent cells and culture medium, as previously described (Castro et al., 2013). The capacity of dual combinations of *Gardnerella* spp. to displace *L. crispatus* pre-adhered to HeLa cells and to also adhere to HeLa cells was evaluated using a previously developed protocol, with several modifications (Castro et al., 2013). After washing the 24-well tissue culture plates, one mL of *L. crispatus* at 5 × 10^7^ CFU/mL was added to each of the wells. After 4 h of incubation the media was removed, and the wells were washed with one mL of 1 × PBS. Then *L. crispatus* was challenged with 500 µL of each of the four *Gardnerella* spp. at 1  × 10^8^ CFU/mL, in dual combinations. These mixtures were also added to the HeLa cells without *L. crispatus*, and the plates were incubated for 30 min. After incubation, all the wells were washed with one mL of 1 × PBS. To detach all of the cell culture, including the adherent bacteria, 100 µL of Trypsin-EDTA (Sigma-Aldrich) was added and the well plates were incubated for 5 min at 37 °C and in 5% CO_2_. Next, 900 µL of PBS 1 × was added to the wells to stop the trypsinization. All of the wash steps were uniformly performed across the assays. Two controls were included. To determine the ability of each *Gardnerella* spp. to adhere to the HeLa cells, one mL of each species at 1 × 10^8^ CFU/mL was added to each well containing only HeLa cells. To determine the ability of each *Gardnerella* spp. to displace pre-adhered *L. crispatus,* one mL of each species at 1 × 10^8^ CFU/mL was added to wells containing *L. crispatus* adhered to the HeLa cells, prepared in the same way as described for the test assays. All assays were performed three independent times, with technical duplicates.

### Construction of calibration curves for quantitative Polymerase chain reaction (qPCR)

The calibration curves for quantitative polymerase chain reaction (qPCR) were constructed, as previously described (Lameira et al., 2024). The biomass of each strain was resuspended in 1 × PBS. The *L. crispatus* suspension was sonicated (Ultrasonic processor Cole Parmer) three times for 10 s at 40% with 10 s intervals, on ice, between cycles. The optical density (OD) at 620 nm of each suspension was adjusted to a concentration of 1 × 10^8^ CFU/mL, and serial dilutions ranging from 10^8^ to 10^4^ were performed. 100 µL of *E. coli* at 1 × 10^8^ CFU/mL was added to each dilution of target bacteria, as an exogenous control, to normalize extraction efficiency. In addition, 100 µL of HeLa cells suspension was also added to each dilution. The HeLa cells suspension was added to each dilution of the calibration curves to account for any potential loss of efficiency in the extraction and quantification process that may have occurred with the adhesion assay samples. Each suspension was centrifuged at 16,000 g for 10 min at 4 °C. The supernatant was discarded, and the pellet was frozen at −20 °C overnight for further gDNA extraction.

### Genomic DNA (gDNA) extraction and bacterial quantification by qPCR

gDNA was extracted from pellets using a commercial Blood & Tissue Kit (Qiagen, Hilden, Germany), following the manufacturer’s instructions, with some modifications. Briefly, the pellets were re-suspended in 300 µL of 1 × PBS and 300 µL of buffer AL was added to the mixture. In the final step, DNA was eluted in DNAse free water. Genomic DNA concentration was measured using the Nanodrop ND-One-W (Thermo Scientific). Bacterial quantification was performed by qPCR, as previously described (Lameira et al., 2024). All runs were performed in a CFX96 thermal cycler (Bio-Rad, Hercules, CA, USA), using the following cycle parameters: 95 °C for 3 min, followed by 40 cycles of 95 °C for 5 s and 60 °C for 20 s. The qPCR amplifications were conducted in 10 µL reaction mixtures containing five µL of Xpert Fast SYBR Master Mix (Grisp, Porto, Portugal), one µL of primer mixture (10 µM), two µL of DNase-free water, and two µL of a 1:40 dilution of extracted gDNA. The software calculated the cycle threshold (Ct), with the baseline threshold being established using an inter-run calibrator in every run. Any Ct value ≤35 was considered positive. Normalization of the qPCR results was achieved through the equation, where ΔCt corresponds to Δ cycle threshold. The target species corresponded to *G. vaginalis*, *G. piotii*, *G. leopoldii*, *G. swidsinskii*, and *L. crispatus* and the control species corresponded to *E. coli*. Calibration curves were generated using the above equation, with ΔCt values plotted on the *y*-axis and known bacterial concentrations (CFU/mL) on the *x*-axis. The bacterial concentration was determined using the generated calibration curves, with the formula, where corresponds to the bacterial concentration and A and B are values given by the generated calibration curve. Primer efficiency (E) was determined from the slope of a standard curve, constructed with 10-fold serial dilutions of each target bacteria, E = [10^(−1/(slope))^ –1] × 100 (Lameira et al., 2024). The qPCR primers used are presented in Table 1. All sets of primers used were obtained from other studies (Collins et al., 2018; Ribeiro et al., 2019; Latka et al., 2022). The GenBank accession numbers for the genes used to design the primers were 45576854 (*G. vaginalis*), 97755823 (*G. leopoldii),* 95681794 (*G. swidsinskii*), V00296.1 (*E. coli*), and LC065039.1 (*L. crispatus*). There was no information on the gene ID for the *G. piotii* DNAg gene, but the accession number of the strain used to design its primers was CP180510.1. The qPCR runs were done three independent times, with technical duplicates. The main steps of the experimental protocol are depicted in Fig. S1.

**Table 1:** Primers sequence. Sequence of the primers used in this study, with relevant associated data.

Species	Target gene	Primer sequence (5′–3′)	GC content %	Amplicon size (bp)	Melting temperature (°C)	Reference
*G. vaginalis*	DNAg	TATTATAACTAAAGCTGCTG	30.0	141	81.5	Latka et al. (2022)
		TCGCCACTATAGTCG	53.3			
*G. piotii*	DNAg	AGCTGCTTACGATTATAGT	36.8	162	82	Latka et al. (2022)
		TTACTCATTCTAAGCTTAATAG	27.3			
*G. leopoldii*	DNAg	GATACTGCACTGTATCGA	44.4	139	79.5	Latka et al. (2022)
		CAGTATCAATACCAGCC	46.1			
*G. swidsinskii*	DNAg	ATTTAGTTAGATATTTGGCAA	23.8	95	81	Latka et al. (2022)
		ATAGTCATATATTCCGCGC	42.1			
*L. crispatus*	16S rRNA	AGCGAGCGGAACTAACAGATTTAC	30.0	154	83.5	Collins et al. (2018)
		AGCTGATCATGCGATCTGCTT	53.3			
*E. coli*	lacZ	AGCGAAACCGCCAAGACTGTTA	50.0	135	86	Ribeiro et al. (2019)
		AGCGAGCGGAACTAACAGATTTAC	45.8			

### Analytical sensitivity and specificity

The limit of detection was determined when the calibration curves were constructed, as mentioned above. The lowest DNA concentration of each curve was the limit of detection of our assays. Regarding specificity, a BLAST search was performed for every pair of primers and each bacterium used in this study, to determine if the primers detected only their specific target. The analytical specificity was also determined by the melting curve of the qPCR results.

### Inhibition testing

To determine the presence of qPCR inhibitors, serial ten-fold dilutions of one gDNA sample from the assays were performed with DNase free water. Then, a qPCR was carried out, targeting the *E. coli* exogenous control. The software calculated the efficiency of the qPCR reaction.

### Statistical analysis

The results obtained from the displacement assays were analyzed using the multiple *t*-test (Holm-Sidak) and the results obtained from the adhesion of *Gardnerella* spp. to the HeLa cells were analyzed using the *t*-test for paired samples. GraphPad Prism 2016 was the software used. A *p*-value ≤ 0.05 was considered statistically significant.

## Results

### Calibration curves

**Figure 1:**
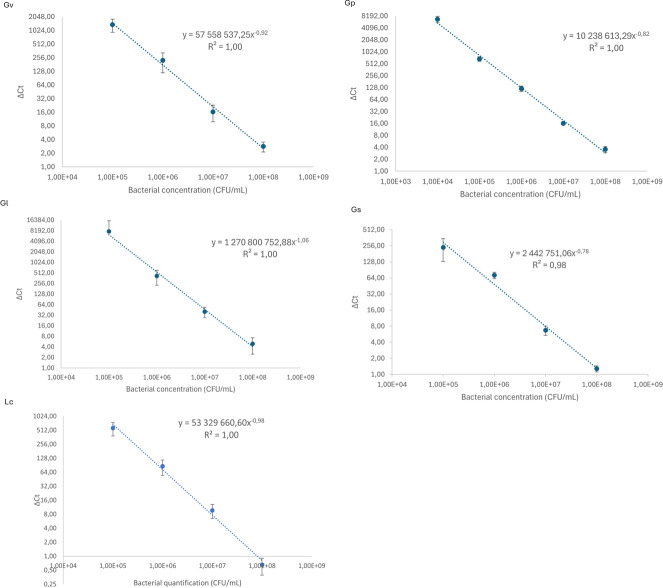
Calibration curves to relate cycle threshold detection (Δ Ct) and bacterial load quantification. Each dot represents the average of three independent assays and the standard deviation.

We started by constructing qPCR calibration curves, depicted in Fig. 1, to quantify the different *Gardnerella* spp. and *L. crispatus* in our adhesion assays. As shown in Fig. 1, all calibration curves presented a linearity of (R^2^) of ≥ 0.98.

### Adhesion of pairwise combinations of *Gardnerella* spp. on HeLa cells

**Figure 2:**
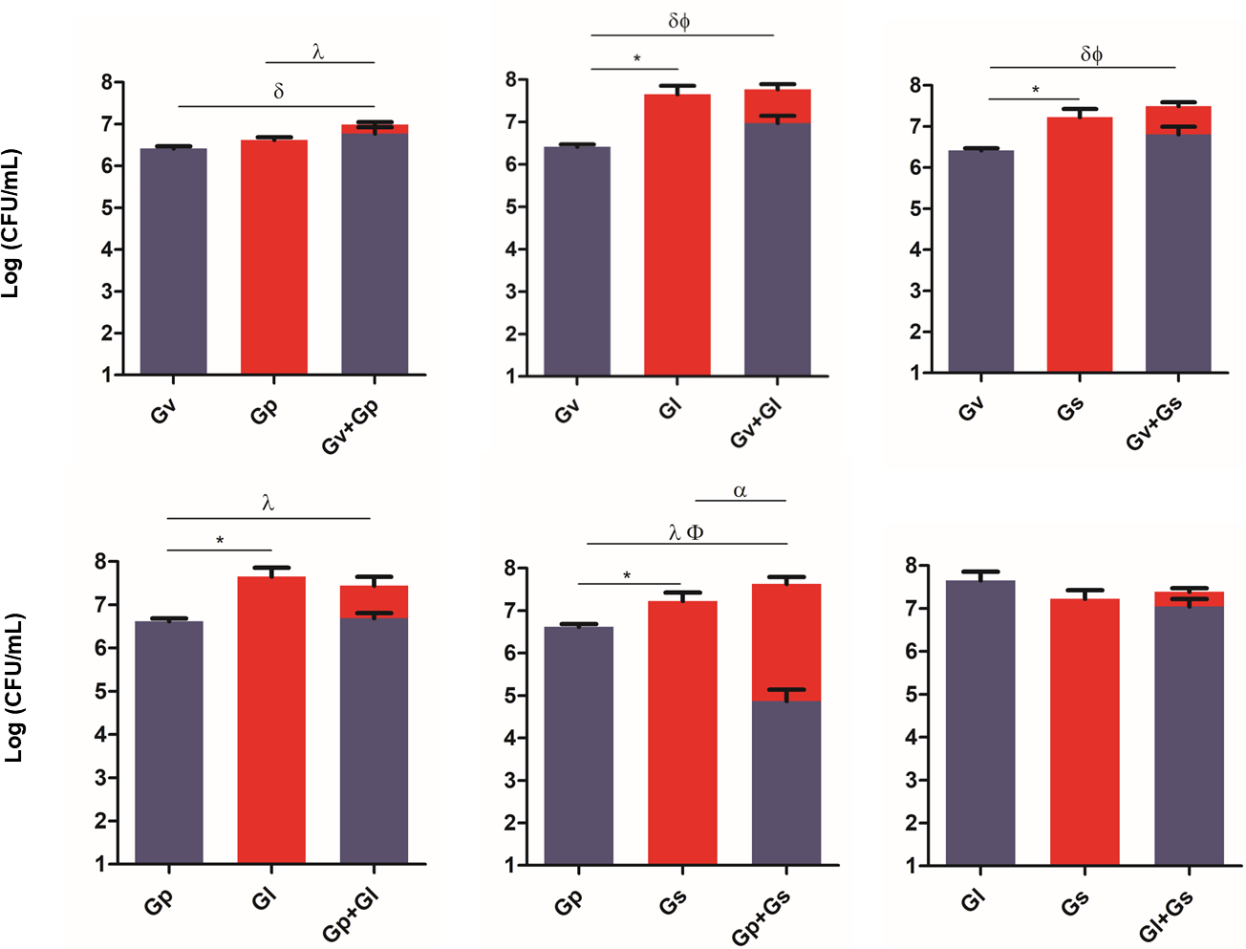
Adhesion of dual combinations of *Gardnerella* spp. to HeLa cells. The bars represent the *Gardnerella* spp. concentration alone or in dual combinations that adhered to HeLa cells. The bars also represent the mean and the standard deviation of three independent experiments. An asterisk (*) represents statistical significance between *Gardnerella* spp. alone; *δ* represents statistical significance between *G. vaginalis* (Gv) alone and the mix; *λ* represents statistical significance between *G. piotii* (Gp) alone and the mix; *ɛ* represents statistical significance between *G. leopoldii* (Gl) alone and the mix; α represents statistical significance between *G. swidsinskii* (Gs) alone and the mix; Φ represents statistical significance between Gv alone and Gv in the mix and β represents statistical significance between Gs alone and Gs in the mix (*t*-test, paired samples, *p* ≤ 0.05). Gv, *G. vaginalis*; Gp, *G. piotii*, Gl, *G. leopoldii* and Gs, *G. swidsinskii*.

To evaluate the ability of the selected *Gardnerella* spp. to adhere to and colonize HeLa cells, and to explore potential synergistic effects in the initial adhesion process, we quantified their adhesion in single and pairwise combinations after a 30-min challenge. As shown in Fig. 2, a variable synergistic effect was observed, with most of the combinations tested resulting in significantly (*p* ≤ 0.05) higher adhesion numbers in the dual-species challenges compared to the single-species challenges. The exception was *G. piotii* cultured with *G. leopoldii*, whereby, their combination resulted in a total bacterial colonization lower than the *G. leopoldii* colonization alone. The combination of *G. vaginalis* and *G. leopoldii* showed the highest synergistic effect on the initial adhesion to HeLa cells, as it was 13 times more efficient than the *G. vaginalis* and *G. piotii* pair (the least efficient dual-species combination). The significant statistical differences observed in this experiment are highlighted in Table S1.

### Displacement of *L. crispatus* by pairwise combinations of *Gardnerella* spp.

**Figure 3:**
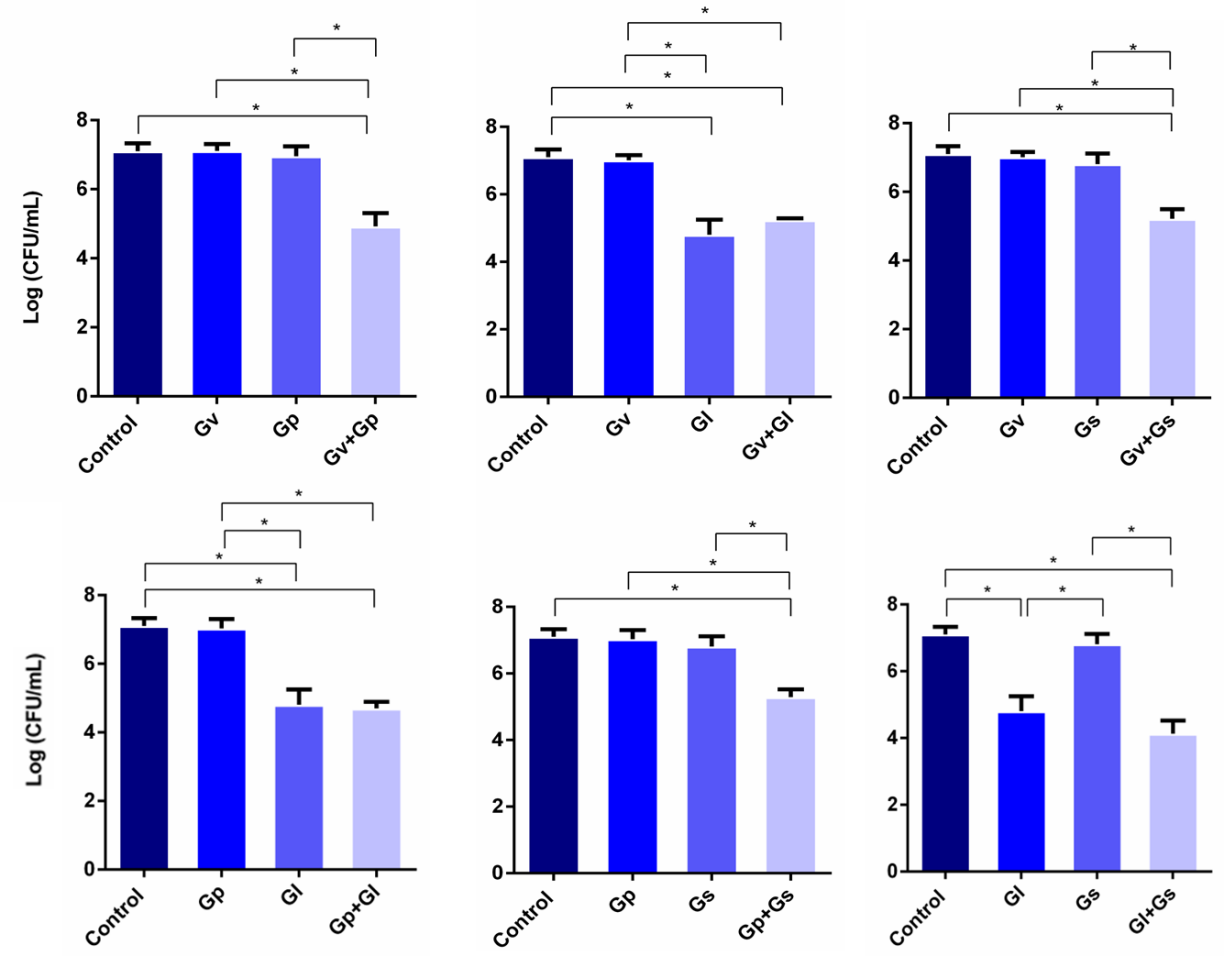
Displacement of *L. crispatus.* by dual combinations of *Gardnerella* spp. The bars represent *L. crispatus* concentrations before (Control) and after being challenged with *Gardnerella* spp. The bars also represent the mean and the standard deviation of three independent experiments. An asterisk (*) represents statistical significance (multiple *t*-test, *p* ≤ 0.05). Gv, *G. vaginalis*; Gp, *G. piotii*, Gl, *G. leopoldii* and Gs, *G. swidsinskii*.

After assessing the potential synergistic effects between the different *Gardnerella* spp., we performed a second experiment, in which *L. crispatus* was first allowed to colonize HeLa cells for 4 h, after which the single and dual-species challenges were repeated. This experiment aimed to simulate a possible external challenge to the vaginal epithelium of a healthy woman colonized by *L. crispatus*. The main objective was to observe if any synergistic effects could be found when attempting to displace the pre-adhered *L. crispatus*. As shown in Fig. 3, most of the dual-species combinations of *Gardnerella* spp. tested resulted in a higher ability to displace the pre-adhered *L. crispatus*, in comparison with the single-species challenges. Also, it was observed that *G. leopoldii* was the tested species with the highest innate ability to displace *L. crispatus* from the HeLa cells. Interestingly, the observed effect was combination-dependent, with the combination of *G. leopoldii* and *G. swidsinskii* having the highest ability to reduce *L. crispatus* colonization. Compared to the less efficient pair *G. piotii* and *G. swidsinskii*, the *G. leopoldii* and *G. swidsinskii* pair was 10 times more efficient in displacing *L. crispatus*. The significant statistical differences observed in this experiment are highlighted in Table S2.

### Adhesion of pairwise combinations of *Gardnerella* spp. on HeLa cells covered with *L. crispatus*

**Figure 4:**
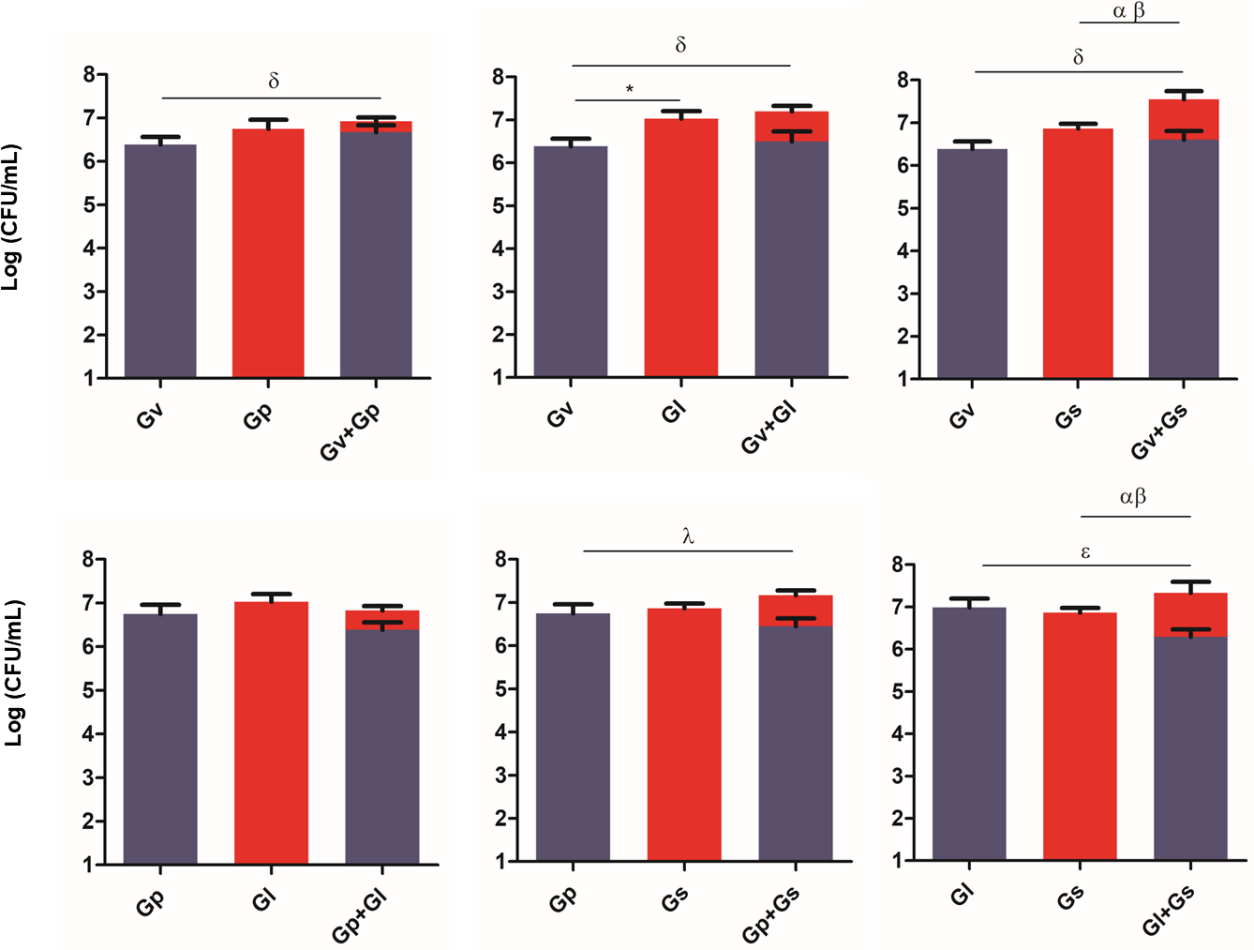
*Gardnerella* spp. dual combination adhesion to HeLa cells with pre-adhered *L. crispatus*. The bars represent the *Gardnerella* spp. concentration alone or in dual combinations that adhered to HeLa cells. The bars also represent the mean and the standard deviation of three independent experiments. An asterisk (*) represents statistical significance between *Gardnerella* spp. alone; *δ* represents statistical significance between Gv alone and the mix; *λ* represents statistical significance between Gp alone and the mix; *θ* represents statistical significance between Gp alone and Gp in the mix; *ɛ* represents statistical significance between Gl alone and Gl in the mix; α represents statistical significance between Gs alone and Gs in the mix and β represents statistical significance between Gs alone and Gs in the mix (*t*-test, paired samples, *p* ≤ 0.05). Gv, *G. vaginalis*; Gp, *G. piotii*, Gl, *G. leopoldii* and Gs, *G. swidsinskii*.

Parallel to determining *L. crispatus* quantification after the *Gardnerella* spp. challenges, we also quantified how many *Gardnerella* spp. were able to remain adhered to the HeLa cells after the challenges. Not surprisingly, when comparing Fig. 4 with Fig. 2, we verified that in most cases, in the presence of *L. crispatus*, the *Gardnerella* spp. pairs concentration was significantly lower than in its absence. The only exception was the dual culture of *G. vaginalis* and *G. swidsinskii*, suggesting that unique interactions between these two species may allow them to overcome *L. crispatus* defenses more effectively. Interestingly, the *G. piotii-G. leopoldii* pair adhered less than *G. leopoldii* alone, although the difference was not significant. In contrast, the combination of *G. vaginalis* and *G. swidsinskii* showed the highest ability to adhere to HeLa cells in the presence of *L. crispatus.* The significant statistical differences observed in this experiment are highlighted in Table S3.

## Discussion

Despite many years of extensive research on the pathogenesis of BV, its etiology is not yet fully determined, hindering the development of improved diagnostic, treatment, and prevention measures (Redelinghuys et al., 2020). As such, continued investment in the study of BV pathogenesis is essential. Here we investigated the interactions of four *Gardnerella* spp. (in dual combinations) on their ability to adhere to HeLa cells and also displace pre-adhered *L. crispatus. L. crispatus* is frequently used as a model to study BV because it is the dominant protective vaginal *Lactobacillus* spp. in most women of childbearing age, harboring antimicrobial properties (Witkin & Linhares, 2017). Furthermore, women with a vaginal microbiota dominated by *L. crispatus* are generally at lower risk of developing BV, and its presence during BV treatment is also associated with better outcomes (Chee, Chew & Than, 2020). Previous studies have demonstrated that *Gardnerella* spp. isolated from BV cases are able to significantly displace *L. crispatus* (Castro et al., 2013; Castro et al., 2015). Interestingly, in this study, when we tested dual-species *Gardnerella* combinations, we observed synergism in most cases. However, the combination that showed the greatest ability to displace *L. crispatus* was not the same pair that demonstrated the greatest capacity to adhere to HeLa cells. This suggests that the ability of *Gardnerella* spp. to displace *L. crispatus* may not be solely dependent on their capacity to adhere to epithelial cells, but also on other factors, such as competitive exclusion (Ghoul & Mitri, 2016) or modulation of host immune responses (Verspecht et al., 2021). It is important to highlight that our adhesion assay measured post-wash recovery of bacteria, and that this readout reflected the sum of all adhesion forces (electrostatic, hydrogen bonding, van der Waals, hydrophobic, and hydrophilic interactions) (Elbourne et al., 2019). These assays reported the net number of bacteria remaining after a defined wash procedure, and therefore cannot intrinsically distinguish between direct competition for binding sites on the epithelial monolayer or weakened adhesion forces leading to differential wash-off from the plastic, which is a technical limitation (Song, Koo & Ren, 2015). However, it is important to emphasize that all experiments were subjected to identical wash stringencies. Therefore, any differences in *L. crispatus* recovery must have arisen from changes in its adhesion strength induced by the presence (and specific composition) of the *Gardnerella* spp., rather than by variable wash conditions.

Our experiments support the feasibility of our hypothesis and are consistent with data presented in other studies demonstrating the potential synergism of *Gardnerella* spp. *in vivo*, with BV-positive participants having multiple species of *Gardnerella* detected in their vaginal specimens (Balashov et al., 2014; Munch et al., 2024). The fact that co-existence of multiple *Gardnerella* spp. may facilitate BV pathogenesis can be explained by significant differences in the accessory genome of these species, which can result in complementary virulence (Vaneechoutte et al., 2019). Nevertheless, it has been reported that antagonistic interactions can occur between different *Gardnerella* spp., wherein reduced growth rates were observed (Khan, Voordouw & Hill, 2019). While this prior study shows different ecological interactions to the ones reported here, it should be highlighted that both studies are fundamentally different in their methodologies. The conditions we tested did not include bacterial growth in rich media, a condition very different from what occurs in the vaginal niche.

Another limitation of our study was the use of HeLa cells, a tumoral cell line, instead of a normal epithelial cell line. We used HeLa cells in our experiments as they have fast growth, good adherence, and high viability, ideal characteristics for repetitive and reproducible assays. Because they are a stable and well-characterized line, consistent results between different laboratories can be ensured (Lih Yuan et al., 2023). In addition, we wanted to compare these results with our previous studies (Castro et al., 2013; Castro et al., 2015). However, we are aware that HeLa cells have reduced expression of certain receptors, namely, Toll-like receptors, which are essential for the recognition of microorganisms and the initiation of a host inflammatory response, when compared to normal epithelial cells (Zhao et al., 2023). Furthermore, our study was limited by the use of pair-wise interactions, the use of one strain per species, and a single methodological approach. While we acknowledge that strain-to-strain variation may exist, our goal was to test an initial specific hypothesis. Indeed, we have demonstrated that synergistic interactions between different *Gardnerella* spp. provide a plausible explanation for the *in vivo* co-colonization of multiple *Gardnerella* spp., supporting our initial hypothesis.

## Conclusions

Overall, our findings support the notion that different *Gardnerella* spp. contribute to successful initial colonization of the vaginal epithelium, highlighting the critical role of *Gardnerella* spp. as early colonizers in the pathogenesis of BV. We do not claim direct clinical extrapolation but instead propose a plausible mechanism, namely cooperative adhesion among *Gardnerella* spp., that may help to explain *in vivo* patterns observed in BV. Nevertheless, our results also support the notion that *L. crispatus* can have a protective effect against *Gardnerella* spp., since in most of the dual-species combinations tested, there was a decrease in *Gardnerella* spp. concentration in its presence. However, there may be a breaking point wherein the concentration of *L. crispatus* is no longer able to prevent *Gardnerella* spp. overgrowth, leading to the onset of BV. This is of relevant importance since the initial adhesion to epithelial cells is a crucial step in BV development. A further understanding of the dynamics of this breaking point is crucial for developing novel treatment and prevention strategies aimed at restoring and maintaining a healthy vaginal microbiota.

## Supplemental Information

10.7717/peerj.20076/supp-1 Supplemental Information 1 MIQE guidelines checklist

10.7717/peerj.20076/supp-2 Supplemental Information 2 Supplemental figure and tables

10.7717/peerj.20076/supp-3 Supplemental Information 3 Raw Data Values of cycle threshold of each sample used in this paper, for different primers (species). With those values we constructed the calibration curves and also the concentration of each species after the adhesion assays. The concentration values allowed us to statistically analyze the observed differences.

10.7717/peerj.20076/supp-4 Supplemental Information 4 Publication license

## Additional Information and Declarations

### Data Availability

The following information was supplied regarding data availability: Data is available in the Supplemental Files.
