# Supplementary material for: Gardnerella species exhibit synergy in their ability to displace Lactobacillus crispatus adhered to HeLa cells
Source: PeerJ. 2025 Nov 5;13:e20076. doi: 10.7717/peerj.20076 (PMC12595946; doi:10.7717/peerj.20076)
Supplement: Supplemental Information 2 [file peerj-13-20076-s002.pdf]

Supplementary material

***Gardnerella* species exhibit synergy in their ability to displace *Lactobacillus crispatus* adhered to HeLa cells**

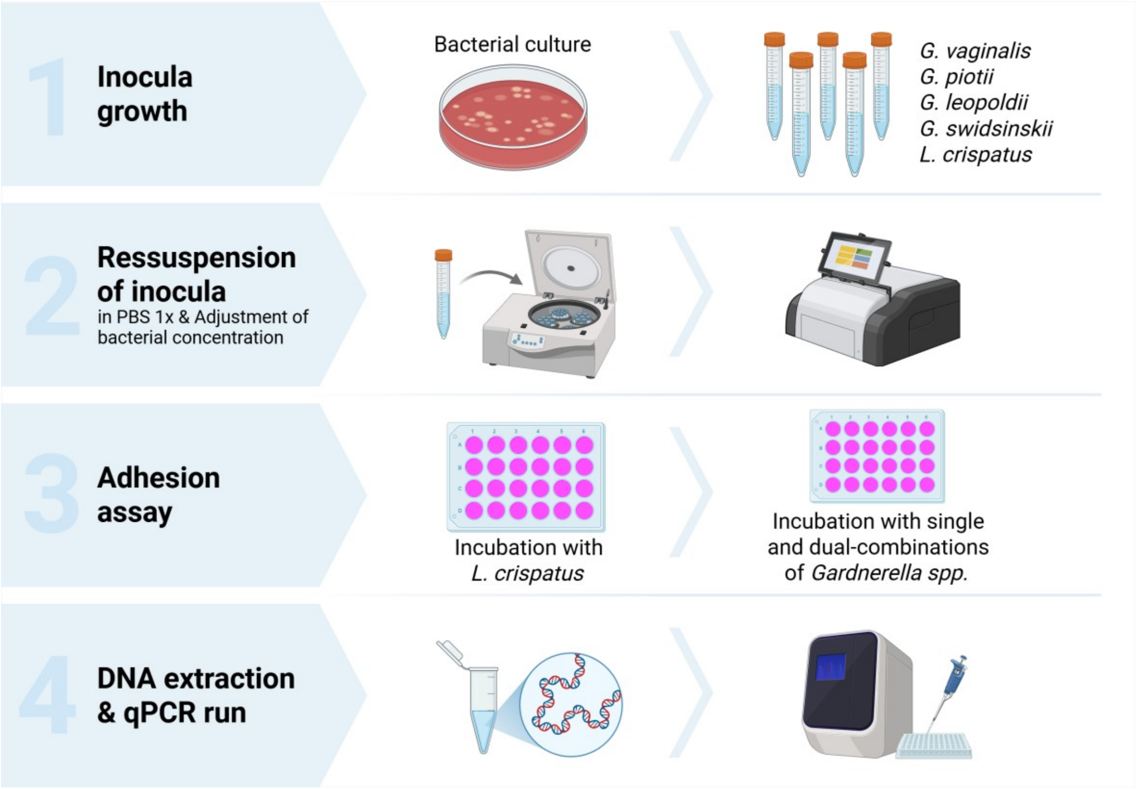

Supplementary Figure 1. Description of the main steps of the experimental workflow.

Supplementary Table 1. Statistically significant difference between the tested groups in Figure 2

**Adhesion of dual combinations of *Gardnerella* spp. to HeLa cells (Figure2)**	
Sets	Statistical difference
Gv vs Gp	No
Gv vs Gv+Gp	Yes
Gp vs Gv+Gp	Yes
Gv vs Gl	Yes
Gv vs Gv+Gl	Yes
Gl vs Gv+Gl	No
Gv vs Gs	Yes
Gv vs Gv+Gs	Yes
Gs vs Gv+Gs	No
Gp vs Gl	Yes
Gp vs Gp+Gl	Yes
Gl vs Gp+Gl	No
Gp vs Gs	Yes
Gp vs Gp+Gs	Yes
Gs vs Gp+Gs	Yes
Gl vs Gs	No
Gs vs Gl+Gs	No
Gs vs Gl+Gs	Yes

Supplementary Table 2. Statistically significant difference between the tested groups in Figure 3.

**Displacement of *L. crispatus* by dual combinations of *Gardnerella* spp. (Figure 3)**	
Control vs Gv	No
Control vs Gp	No
Control vs Gl	Yes
Control vs Gs	No
Control vs Gv+Gp	Yes
Control vs Gv+Gl	Yes
Control vs Gv+Gs	Yes
Control vs Gp+Gl	Yes
Control vs Gp+Gs	Yes
Control vs Gl+Gs	Yes
Gv vs Gp	No
Gv vs Gl	Yes
Gv vs Gs	No
Gp vs Gl	Yes
Gp vs Gs	No
Gl vs Gs	Yes
Gv vs Gv+Gp	Yes
Gv vs Gv+Gl	Yes
Gv vs Gv+Gs	Yes
Gp vs Gv+Gp	Yes
Gp vs Gp+Gl	Yes
Gp vs Gl+Gs	Yes
Gl vs Gv+Gl	No
Gl vs Gp+Gl	No
Gl vs Gl+Gs	No
Gs vs Gv+Gs	Yes
Gs vs Gp+Gs	Yes
Gs vs Gl+Gs	Yes

Supplementary Table 3. Statistically significant difference between the tested groups in Figure 4.

***Gardnerella* spp. dual combination adhesion to HeLa cells with pre-adhered *L. crispatus* (Figure 4)**	
Sets	Statistical difference
Gv vs Gp	No
Gv vs Gv+Gp	Yes
Gp vs Gv+Gp	No
Gv vs Gl	Yes
Gv vs Gv+Gl	Yes
Gl vs Gv+Gl	No
Gv vs Gs	No
Gv vs Gv+Gs	No
Gs vs Gv+Gs	Yes
Gp vs Gl	No
Gp vs Gp+Gl	No
Gl vs Gp+Gl	No
Gp vs Gs	No
Gp vs Gp+Gs	Yes
Gs vs Gp+Gs	No
Gl vs Gs	No
Gs vs Gl+Gs	Yes
Gs vs Gl+Gs	Yes
